# Towards Understanding the Role of Surface Gas Nanostructures: Effect of Temperature Difference Pretreatment on Wetting and Flotation of Sulfide Minerals and Pb-Zn Ore

**DOI:** 10.3390/nano10071362

**Published:** 2020-07-12

**Authors:** Yuri Mikhlin, Anton Karacharov, Sergey Vorobyev, Alexander Romanchenko, Maxim Likhatski, Svetlana Antsiferova, Svetlana Markosyan

**Affiliations:** Institute of Chemistry and Chemical Technology, Krasnoyarsk Science Center of the Siberian Branch of the Russian Academy of Sciences, Akademgorodok, 50/24, 660036 Krasnoyarsk, Russia; karacharov@icct.ru (A.K.); yekspatz@ya.ru (S.V.); romaas82@mail.ru (A.R.); lixmax@icct.ru (M.L.); sveta_a@icct.ru (S.A.); obog@icct.ru (S.M.)

**Keywords:** surface nanobubbles, hydrophobicity, oxidation, flotation, lead sulfide, zinc sulfide, Pb-Zn ore

## Abstract

Surface nanobubbles at hydrophobic interfaces now attract much attention in various fields but their role in wetting-related phenomena is still unclear. Herein, we report the effect of a preliminary contact of “hot” solids with cold water previously proposed for generation of surface nanobubbles, on wettability of compact materials and flotation of particulate galena (PbS), sphalerite (ZnS), and Pb-Zn sulfide ore. Atomic force microscopy was applied to visualize the nanobubbles at galena crystals heated in air and contacted with cold water; X-ray photoelectron spectroscopy was used to characterize the surface composition of minerals. Contact angles measured with the sessile drop of cold water were found to increase when enhancing the support temperature from 0 to 80 °C for sphalerite and silica, and to pass a maximum at 40–60 °C for galena and pyrite (FeS_2_) probably due to oxidation of sulfides. The temperature pretreatment depressed the recovery of sulfides in collectorless schemes and improved the potassium butyl xanthate-assisted flotation both for single minerals and Gorevskoye Pb-Zn ore. The results suggest that the surface nanobubbles prepared using the temperature difference promote flotation if minerals are rather hydrophobic and insignificantly oxidized, so the addition of collector and activator (for sphalerite) is necessary.

## 1. Introduction

Nanoscale surface gas-like structures, which often, but not exclusively, have the shape of a spherical segment and are referred to as “nanobubbles”, attract a great deal of attention in recent years. These species are believed to be ubiquitous on hydrophobic surfaces and crucially influence many properties of solid/aqueous interfaces [1,2,3,4,5,6], being, for example, in many cases responsible for long-range capillary attraction (so-called hydrophobic forces) [7,8,9,10,11,12]. The mechanisms of formation and unusual stability, characteristics of the nanobubbles and their role in wetting, reactivity and other processes on the interfaces are insufficiently understood. Up to now, the overwhelming majority of studies have been performed using a solvent exchange technique for the nanobubble preparation (a sample is conditioned in ethanol and then in water). Atomically smooth highly oriented pyrolytic graphite (HOPG) is commonly used as a support, and atomic force microscopy and spectroscopy (AFM/AFS) in water is utilized for visualization and examination of nanobubbles (see, for example [3,4,5,6]). Employing these techniques, however, seems difficult or impossible for real materials, especially particulate ones. 

One should expect that nanoscale gas domains can arise in hydrophobic minerals and affect their behavior in nature and mineral processing; first of all, particle–bubble interaction in air-saturated froth flotation slurries [13,14,15,16,17]. However, only in few works the nanobubbles have been detected experimentally under conditions related to the flotation processes. Hampton and Nguyen [16] have discussed a potential role of gaseous domains accumulated at the interface of graphite and aqueous solutions for froth flotation of coal; the common AFM and solvent exchange methods on HOPG were used in the experiments. Mikhlin et al. [18] found surface nanobubbles on HOPG and galena (PbS) pre-treated with potassium butyl xanthate solution using AFM/AFS, that for the first time involved a real mineral and flotation collector. Moreover, quasi in situ X-ray photoelectron spectroscopy (XPS) studies of the particulate sulfide minerals reacted with xanthate in the slurries, centrifuged and then fast-frozen [19,20], revealed charging effects attributed to cavities separating the hydrophobized mineral particles which may be signatures of gas structures arising on the surfaces under the flotation-related conditions. Owens and co-workers [21,22] have imaged, with non-contact atomic force microscopy, surface nanobubbles on polished cross sections of dolomite and rare earth fluorcarbonate mineral synchysite treated with flotation collectors inducing hydrophobicity of the minerals. Xing et al. [23] have surveyed the hydrophobic force in particle–bubble attachment and concluded that the role of nanobubbles still remains to be identified. 

Several researchers proposed a temperature difference (TD) method to produce nanobubbles [5,24,25,26]. In particular, An et al. [26] have reported a technique for simple and reproducible preparation of surface nanobubbles stable for several days without additional solvents and reagents by depositing cold water (e.g., 4 °C) on HOPG substrates heated to 40–80 °C. The nucleation of nanobubbles is caused by a lower solubility of gases in the heated water layer closest to the hot substrate than in bulk water. In the current research, we applied this method for generation of surface nanobubbles on galena (PbS) and sphalerite (ZnS) including particulate minerals and a Pb-Zn sulfide ore in order to elucidate the influence of the temperature difference between the “hot” solid and “cold” water and tentative surface gas domains on hydrophobicity and flotation of the minerals. Such a study is also of interest for understanding the mechanisms behind the wetting behavior of material surfaces and coatings in many other applications. 

## 2. Materials and Methods

### 2.1. Materials

Single crystals of natural galena (Taymyr, Russia) were obtained from Geological Museum of Central Siberia (Krasnoyarsk, Russia); fresh fractured surfaces of about 3 mm × 4 mm of the mineral were employed in AFM experiments, as described in detail elsewhere [16,23]. HOPG specimens (ZYH grade) purchased from NT-MDT (Russia) were cleaved directly before experiments. Galena (Gorevskoye deposit, Russia, or Zhairem ore deposit, Kazakhstan) and sphalerite with Fe 2.1 wt.% (Zhairem ore deposit, Kazakhstan) with no visible inclusions of foreign phases used. The mineral plates about 4 mm × 5 mm were abraded using SiC paper (500 grit) and then wiped with wet filter paper and rinsed with water in the contact angle measurement. The minerals were ground in an agate mortar and sieved on standard wire mesh sieves to obtain the fraction less than 75 µm and larger than 44 µm (−75 + 44 µm) about 2 h before the microflotation experiments.

Pb-Zn sulfide ore from Gorevskoye deposit (Krasnoyarsk Territory, Russia) had an average elemental composition, wt.%: Pb 4.4, Zn 2.87, Fe 27.9, S 4.3, Si 5.5, Ca 1.0, Mg 1.0, O 42.2. The main sulfide minerals are galena, pyrrhotite (Fe_7_S_8_), sphalerite, pyrite; gangue minerals are presented by siderite (FeCO_3_), quartz, dolomite ((Mg,Ca)CO_3_), calcite (CaCO_3_), and some others. Ore samples were ground in a steel ball mill to 85% finer than 75 µm at a solid/water/balls weight ratio of 1:0.5:9; some characteristics of the ground ore were reported earlier [24]. Then the solid and liquid phases were separated and heated or cooled in air to the temperatures ranged from 0 to 60 °C as described below (see also Supplementary Materials).

Commercial potassium n-butyl xanthate (KBX) of about 95% purity was recrystallized in acetone and then stored in solid state at 0 °C; aqueous solutions of KBX were prepared before the experiments using deionized water. Other chemicals were of analytical grade and were used as received. Deionized water (Millipore Milli-Q grade) was used to prepare all the solutions. No plastic syringes, tubing, and so forth were utilized for handling the solutions and samples to avoid possible contamination. 

### 2.2. AFM and XPS Characterization 

Atomic force microscopy experiments were carried out using a multimode scanning probe microscope Solver P47 equipped with a piezoelectric tube scanner (NT-MDT, Moscow, Russia) having the nominal lateral resolution of about 0.1 nm. In the study of the temperature difference, galena or HOPG substrates were fastened at a sapphire plate, and heated to the temperature ranging from 30 to 80 °C in air. In a series of experiments, the mineral was preliminarily conditioned in 0.1 mM or 10 mM KBX solutions for 10 min at room temperature and rinsed with water. The sample was placed in an open liquid Ti cell, and the cell was filled with deionized water (1 mL, pH 5.1) or the solution of KBX cooled to a temperature from 0 to 10 °C, and allowed to equilibrate for about 30 min; AFM measurements were conducted after the aqueous phase reached room temperature. AFM images were obtained in the tapping mode (TM-AFM) with rectangular Si cantilever NSG11 (NT-MDT) with the nominal tip diameter of 10 nm, the resonance frequency of about 110 kHz in water and 150 kHz in air and the spring constant of ~10 N/m. The scan rate was 1–2 Hz; no smoothing was applied. The images were obtained at least in 3–4 points for each sample; typically, 3 parallel samples were examined to ensure the reproducibility. 

The state of mineral surfaces, including fractured, polished, and particulate single minerals and Pb-Zn sulfide ores, were characterized using X-ray photoelectron spectroscopy. The spectra presented here were measured with a SPECS spectrometer equipped with a PHOIBOS 150 MCD-9 analyzer (SPECS, Berlin, Germany) at an electron take-off angle of 90° employing Mg Kα radiation (1253.6 eV) at room temperature. The analyzer pass energy was 20 eV for survey spectra and 8 eV for narrow scans. The C 1s peak at 285.0 eV from hydrocarbon contaminations was used as a reference. The surface concentrations of elements were determined employing the survey scans and the empirical sensitivity coefficients. The high resolution spectra were fitted with the Gaussian–Lorentzian peak profiles after the subtraction of the Shirley-type background using a CasaXPS software (version 2.3.16, Casa Software, Teignmouth, UK).

### 2.3. Contact Angle Measurement

Static contact angles reported here were measured using the sessile drop (13 µL) with an OCA-15EC contact angle meter (DataPhysics Instruments, Filderstadt, Germany); the values reported are the average of at least 3 parallel sample measurements with the error within ±2°. Deionized water or aqueous solutions of collectors were cooled down to 0.2 °C in the syringe and needle applying a cryo-thermostat, while a desired temperature of polished mineral plates of galena and sphalerite was set in the range from 0 to 80 °C using an underlying Peltier element of a temperature unit TPC 160 (DataPhysics Instruments).

### 2.4. Flotation Tests

Flotation of single minerals was carried out utilizing 2 g of galena or sphalerite in each experiment in a modified Hallimond tube with air flow at approximately 50 mL/min. The minerals heated to 40 °C were agitated with icy water until reaching a room temperature (about 30 min) followed by flotation (5 min) in water without collector or in 0.1 mM KBX solutions (pH 9) at ambient temperature. The flotation of sphalerite was also performed using 0.01 mM CuSO_4_ + 0.1 mM KBX solution after the temperature difference pre-treatment. 

The experiments on flotation of the Pb-Zn sulfide ore were carried out in a laboratory flotation impeller machine equipped with a 1000 mL cell. The slurry after the drum milling was separated by sedimentation and decantation to a solid residue and supernatant liquid, which were independently heated or cooled to desired temperatures, and mixed again before flotation. The slurry with the weight solid/water ratio of 1 to 5 equilibrated to room temperature was agitated in the cell (1200 rpm) with or without adding KBX collector and other reagents; the total flotation time was 11 min. The flowsheets and reagent schemes based to those operating at Gorevskoye plant are described in detail in Supplementary Materials. The products were filtered, dried, and the concentration of elements was determined using X-ray fluorescent analysis with an Axios advanced Panalytical instrument (Panalytical BV, Almelo, The Netherlands). All the flotation experiments with minerals and the ore were repeated at least three times, with the average data presented.

## 3. Results

### 3.1. Atomic Force Microscopy

**Figure 1:**
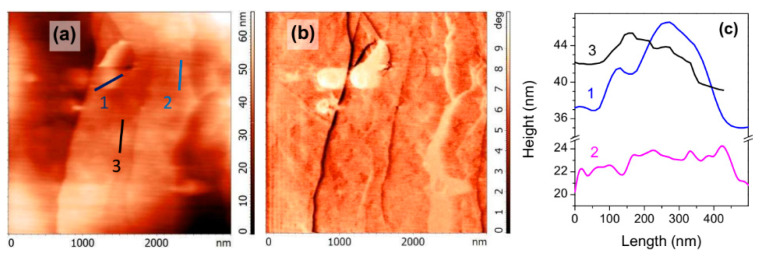
Tapping mode atomic force microscopy (AFM) height (**a**) and phase (**b**) images of highly oriented pyrolytic graphite (HOPG) surface heated to 60 °C and then conditioned in cold water (5 °C) before AFM experiment in ambient water; (**c**)—height profiles along lines at (**a**) image.

As almost all studies on the nanobubble formation, including the effect of TD, have been conducted with a HOPG substrate, and we first checked the applicability of our techniques on HOPG. Figure 1 shows the representative tapping mode AFM images (height and phase) acquired in room temperature water from HOPG that was heated before the contact with cold water. Species arising due to the TD treatment and well discernible both in the height and phase images had the lateral size of 100–300 nm and the height of 5–12 nm which agree with the dimensions of nanobubbles found by other authors [24,25,26] despite some differences in the AFM and sample preparation techniques. In particular, more rigid cantilevers used in the current research could affect apparent bubble dimensions [27]. We observed such nanobubbles in more than 50% of experiments. It seems possible that there also exist other gas structures on HOPG, including “pancakes” of 1–2 nm thick [2,3,4,5,6] and surface gas mono- and multi-layers [28], which are slightly visible (Figure 1, Section 2 and Section 3; see also Figure S1 in Supplementary Materials) or may be invisible in the current experimental setup. On the other hand, submillimeter bubbles undetected with AFM but visible with an optical microscope or the naked eye develop with time at the pre-heated surfaces of HOPG and minerals in cold water, depending upon the temperature regime and pre-treatment with flotation collectors. 

**Figure 2:**
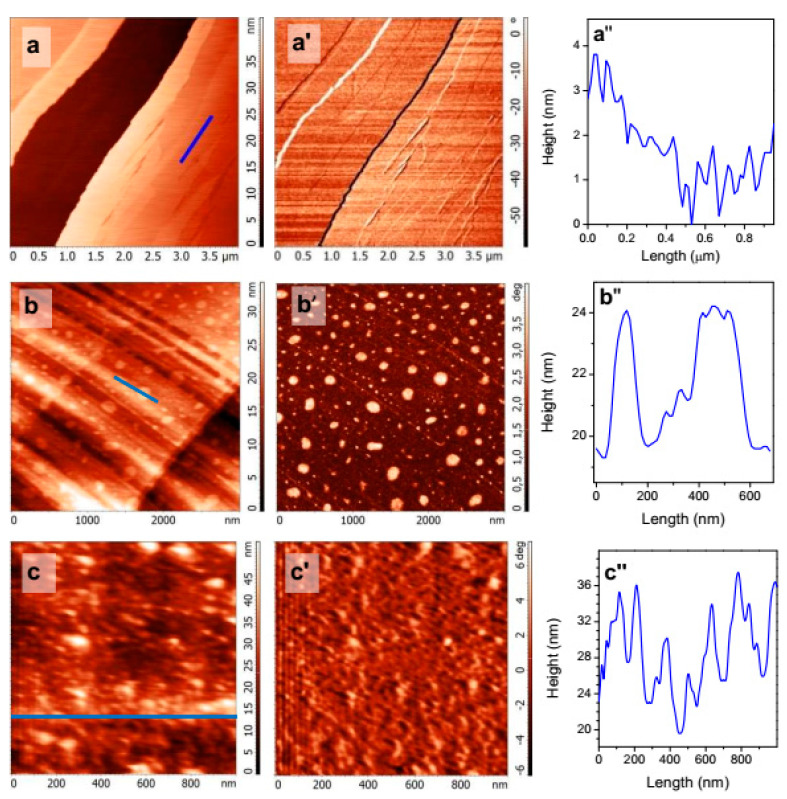
Tapping mode AFM images taken in ambient water from galena surfaces (**a**,**a′**) fractured, (**b**,**b′**) pre-treated using the temperature difference procedure in water (galena (PbS) heated in air to 50 °C and then contacted with water cooled to 5 °C); (**c**,**c′**) heated at 50 °C and then reacted with cold (5 °C) 10 mM potassium n-butyl xanthate (KBX); (**a**–**c**)—height, (**a′**–**c′**)—phase contrast, (**a″**–**c″**)—relief profiles along the blue lines at the height images (**a**–**c**), respectively.

Figure 2 shows AFM images collected in water from freshly fractured surface of galena (a, a′, a″) and those preliminarily heated and then conditioned in cold water. The PbS surfaces are composed of terraces formed by crystal planes (100) and steps from atomic to several nm in the height. At the galena after the TD conditioning (Figure 2(b,b′,b″)), terraces are nanometer-scale rough due to the etch of PbS and oxidation products formed [18,29,30,31,32,33]. The larger entities are believed to be surface nanobubbles, which have the dimensions similar to those observed at HOPG (Figure 1). The nanobubbles previously found on galena reacted with butyl xanthate at room temperature had comparable lateral dimensions and 3–8 nm in height [18]. Neither such reaction products nor contaminations were detected using XPS (see below) and AFM [18,29] under comparable conditions.

The results of AFM study on combined application of the TD and flotation collector KBX treatment are not conclusive because of PbS reactivity (oxidation) at elevated temperatures. Figure 2(c–c″) represents characteristic AFM data for the surface of galena heated and then reacted with cold 10 mM KBX solution prior the AFM experiment. The large number of species with the diameter of 50–200 nm and the height of 2–10 nm can be attributed to surface nanobubbles similar to those observed earlier but also to the products of oxidation of PbS, mainly hydrophilic, and decomposition of xanthate, including lead xanthate [18,29,30,31,32,33,34]. Moreover, in some experiments we observed etch pits of 10–20 nm deep together with increased amount of surface reaction products (Figure S2).

The AFM study demonstrates, therefore, that the TD pretreatment does produce surface nanobubbles, which slightly differ from those formed as a result of the collector adsorption on PbS [18]. After application of both the TD and butyl xanthate treatment, the nanobubbles seem to coexist with the products of PbS reactions. It is important that the nanobubbles maintain after equilibrating the temperatures of galena and water. Nanobubbles were not surely specified in the experiments on sphalerite and pyrite, mostly because of their roughness as we avoided fine polishing and other procedures which may cause severe oxidation and distortion of the surfaces. A more detailed characterization of the gas structures was beyond the scope of this study.

### 3.2. Contact Angle Measurement

**Figure 3:**
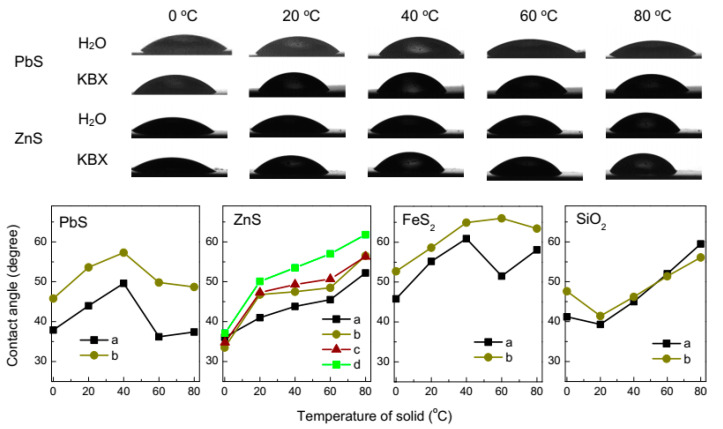
Upper panels: examples of sessile water drops cooled to 0.2 °C at galena and sphalerite plates kept at various temperatures. Lower panels: contact angles measured on the surfaces of PbS, sphalerite (ZnS), FeS_2_ and SiO_2_ (oxidized Si) plates as a function of the temperature of solid: (**a**) rinsed with water and (**b**) reacted with 10 mM potassium n-butyl xanthate solution (KBX) for 10 min. ZnS was also preliminarily reacted with (**c**) 0.1 mM CuSO_4_ solution and then with 10 mM KBX (**d**).

The impact of the temperature difference between a mineral and water, and so possible nanobubbles on surface hydrophobicity was studied using the sessile drop method. Figure 3 represents several examples of the water droplets cooled to 0.2 °C and placed on mineral plates kept at various temperatures, and summary of the average static contact angles. The data show that the wetting generally decreases, and the angles increase, with enhancing the temperature of solid supports. For galena, the contact angle grows as the substrate temperature increases to 40 °C, and then decreases; the maximum persists for the mineral preliminarily hydrophobized with 10 mM KBX solution. Similar maxima at pyrite are shifted towards a higher temperature. The contact angle at more hydrophilic sphalerite steadily increases with temperature both for the mineral polished in air and that activated with aqueous copper sulfate before and after pre-conditioning in 10 mM KBX solution.

The changes of wettability are unlikely to be due to temperature dependence of the surface tension [35,36] and may be tentatively interpreted in terms of the formation of gas structures promoting hydrophobicity of sulfide minerals as their temperature increases. The effect competes with oxidation of sulfides, which makes them more hydrophilic, so the maxima at the plots can be explained by oxidation of galena and pyrite accelerated at higher temperatures [29,30,31,32,33,37,38,39]. This concurs with the behavior of sphalerite that is more resistant to oxidation, and silica becoming less hydrophilic with increasing temperature, while wetting of hydrophobic HOPG (not shown in figures) changes insignificantly. The surface tension of water decreasing with enhancing temperature may, however, contribute to higher wettability nearby 0 °C and above 40–50 °C for galena and pyrite.

### 3.3. X-ray Photoelectron Spectroscopy Characterization

**Figure 4:**
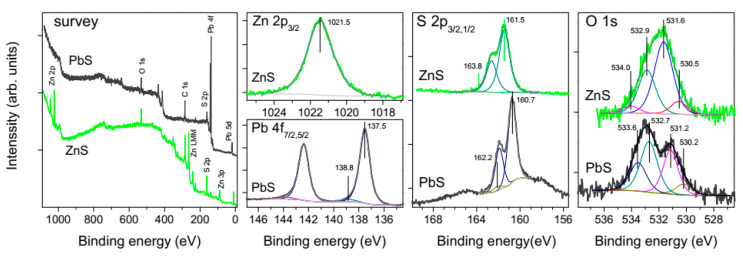
X-ray photoelectron spectra collected from polished and washed plates of natural PbS and ZnS used in the contact angle measurements.

The composition and chemical state of the surfaces of metal sulfides and ores was controlled using XPS analysis of the compact and particulate materials in various stages of the treatment. Figure 4 shows the spectra of the lead sulfide and zinc sulfide plates polished and washed with water as described in Section 2.1, and then utilized in contact angle measurements. The survey spectra confirm the absence of foreign phases, including conceivable Si-bearing lubricant contaminations from plastic cyringes, tubes, fittings, etc. [40], almost stoichiometric composition of PbS and ZnS surfaces, and negligible contents of impurity elements, excepting for Fe (about 2 at.%) in sphalerite. The high-resolution Pb 4f and Zn 2p spectra are characteristic of the metal sulfides [18,19,20,30,37,41] and indicative of low quantities of oxidized metal compounds; for example, the relative intensity of the Pb 4f_7/2_ component at the binding energy (BE) of 138.8 eV from lead oxide or/and hydroxide is as small as 4 rel.%. The S 2p spectra are also typical for PbS and ZnS, and show very minor contributions of disulfide and polysulfide anions (BEs of 162.2 eV and 163.8 eV, respectively), and no S-O species at BEs above *~*166 eV; please note that the S 2p spectral region for PbS is overlapped with the secondary structure from Pb 4f maxima (plasmons, etc.). The O 1s spectra of PbS, which are somewhat different for galena and sphalerite, can be fitted using the peaks at 530.2 eV (most likely, from minor surface PbO), 531.2 eV (OH^−^ groups), 532.7 eV and 533.6 eV, attributable to adsorbed water or/and oxygen in adventitious carbonaceous contaminations. Similar maxima, shifted to higher BEs by 0.2–0.4 eV probably because the Fermi level position in the bandgap of ZnS, were found for sphalerite. The component at 531.5 eV that likely originates from adsorbed OH^−^ ions is, however, notably stronger than its counterpart for PbS. This may be a reason for the lower hydrophobicity of sphalerite. 

The photoelectron spectra insignificantly altered after the treatments of the metal sulfides applied here, and are not shown in Figures. However, it should be taken in consideration that modification of the sulfide surfaces occurs ex situ upon handling the samples and XPS experiment in the ultra-high vacuum, including volatization of water, elemental sulfur, polysulfide anions, dixanthates, and decomposition of solid/water interface and some compounds. The species adsorbed due to the interaction with xanthate collector and other flotation reagents at particulate sulfide minerals [18,19] and initial oxidation of chalcopyrite [42] were studied previously using low-temperature XPS of the fast-frozen specimens. We applied this method for examination of the oxidation of galena, sphalerite, and pyrite; the results to be published elsewhere. Application of the cryo-XPS for characterization of ground ores and compact materials is still problematic. 

### 3.4. Single Mineral Flotation 

**Figure 5:**
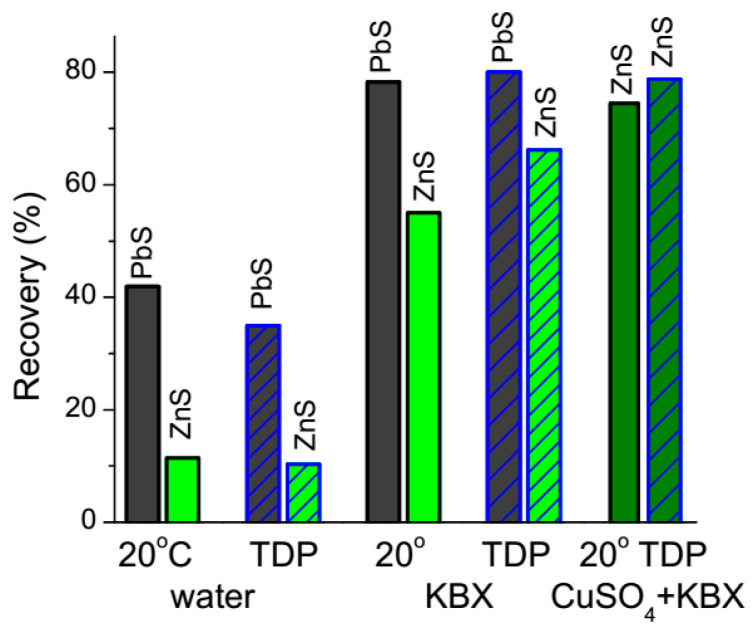
Flotation recoveries of PbS (galena) and ZnS (sphalerite) without (marked as 20 °C) and with the temperature difference pretreatment (TDP, solid heated to 40 °C, water cooled to 2 °C), with 0.1 mM KBX solution, and also with 0.02 mM CuSO_4_ and 0.1 mM KBX solution for sphalerite.

Figure 5 shows the results of microflotation of galena and sphalerite in a Hallimond tube. The recovery under collectorless conditions somewhat decreased for galena and, slightly, for sphalerite preliminarily heated to 40 °C and conditioned in cold water in comparison with the normal flotation at room temperature. Flotability of both minerals notably increased if collector (0.1 mM KBX) was added. In this case, the preliminary TD treatment exerted a small positive effect on the flotation of galena, and the recovery of sphalerite increased more notably. Flotation of sphalerite is further improved using the activation with copper ions, and TD procedure also shows a positive effect. The higher floatability after the pretreatment correlates with the enhanced hydrophobicity of the minerals (Figure 3) and may be due to surface nanobubbles arising at their surfaces (Figure 2), while the negative effect in the collectorless scheme may result from oxidation of sulfides after long contact with water [29,30,31,32,33,37].

### 3.5. Flotation of Pb-Zn Sulfide Ore 

**Figure 6:**
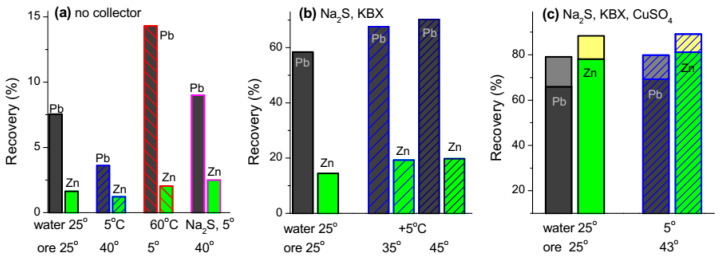
The results of bulk sulfide flotation (recoveries of Pb and Zn) from Gorevskoye Pb-Zn sulfide ore in various reagent scheme with and without the temperature difference pretreatment (the temperatures of aqueous phase and ore are shown at abscissa). (**a**) No collector added: no TD pre-treatment (both water and ore of about 25 °C); ore heated to 40 °C, water cooled to 5°; ore cooled to 5°, water heated to 60 °C; Na_2_S (100 g/t) added to the mill, ore heated to 40 °C, water cooled to 5°. (**b**): Na_2_S (100 g/t) and Na_2_CO_3_ (1000 g/t) were added to the mill, KBX (150 g/t) and frother T-92 (50 g/t) was dosed to flotation cell (pH 9.2, 20 °C), temperature pre-treatment regimes are shown at abscissa. (**c**) Na_2_S (100 g/t) and Na_2_CO_3_ (2000 g/t) were added to the mill, KBX (150 g/t), frother T-92 (50 g/t) and CuSO_4_ (200 g/t) were dosed to the flotation cell, the upper parts of the columns show additional recoveries in one-step tail cleaning.

The experiments on bulk sulfide flotation of the Pb-Zn ore, which separates hydrophobic sulfide minerals from ganguies, were conducted in a collectorless regime using “normal” mill products as a reference in comparison with the experiments in which the ground solid was heated to about 40 °C and mixed with the aqueous phase cooled to +5 °C before the flotation; the residue was cooled and the liquid was heated up to 60 °C. Figure 6 shows (see also Tables S1–S5, Supplementary Materials) that the recovery of Pb increased after the ore treatment with hot water and decreased in the case of cold water. The floatability of sphalerite changed insignificantly. When sodium sulfide was added in order to suppress oxidation of metal sulfides (Figure 6a), we observed “normal” effect of the temperature difference, that is, the increased flotation of minerals preliminarily contacted with cold water. We believe that the oxidation of galena upon milling and heating play the main role, while hot water (and, in a smaller extent, sodium sulfide) removes hydrophilic fines and slimes [24,43] and oxidation products from the mineral surfaces. 

**Figure 7:**
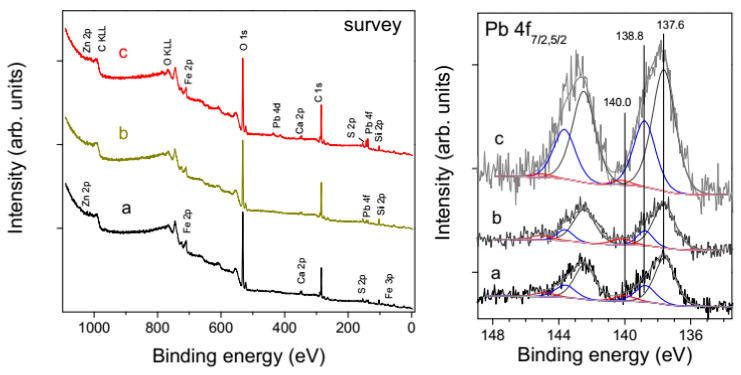
Photoelectron survey and Pb 4f spectra from (**a**) ground Pb-Zn sulfide ore, (**b**) the ground ore treated with 0.1 M Na_2_S solution (10 min); (**c**) the ground ore treated with water heated to 60 °C (10 min).

Figure 7 presents photoelectron survey spectra and the spectra of lead acquired from the Pb-Zn ore ground and then conditioned in 0.01 M Na_2_S solution at room temperature or water at 60 °C; the spectra of S and other elements are not given to avoid confusion with the contributions of different minerals. One can see that the intensities of Fe and O lines (survey spectra) and maxima from lead oxide and hydroxide at the Pb 4f_7/2_ binding energy of 138.8 eV somewhat decrease while PbS signal at 137.5 eV increases (from 66 to 73 rel.%) after the reactions with sulfide ions (spectra b). Moreover, the Pb 4f and S 2p spectra measured after the hot water treatment become about two times stronger, probably due to elimination of surface ultrafines [43], in agreement with the previous XPS studies on galena and Gorevskoye ore [29,30]. 

The next series of tests were performed with adding KBX collector but without sphalerite activation with CuSO_4_; this increases the recovery of lead while that of zinc remains low. The preliminary heating the solid and cooling the aqueous phase essentially improve both the recovery of lead (from 58% to about 70%) and zinc (14.5% to 19%) and the quality of the bulk sulfide concentrate, with the contents of Pb and Zn growing from 31 to 40 wt.%, and from 6 to 7 wt.%, respectively (Supplementary Materials). At last, the bulk flotation experiments were carried out using the complete reagent scheme including addition of CuSO_4_ and frother T-92, and one-stage cleaning of the tails. The flotation of zinc sharply increased with the activator, and a positive effect of the temperature difference pre-conditioning (“hot” ore and cold water) was small but still observable both for lead and zinc recovery.

## 4. Discussion

This study demonstrated that the preliminary contact of “hot” metal sulfides and other solids with colder water promoted a generation of surface nanobubbles, and increased the hydrophobicity of solids and recovery of lead and zinc in froth flotation of the sulfide minerals and ores. The nanobubbles remain at the surfaces after the temperatures were equilibrated, in agreement with the findings reported previously for a model HOPG substrate [24,25,26]. The morphologies of the surfaces seem somewhat different from those formed due to the reaction with hydrophobizing n-butyl xanthate solutions [18] and combination of the TD and xanthate pretreatments. 

The gas nanobubbles obtained with the temperature difference method do not ensure high enough hydrophobicity and floatability, and their effect is pronounced in the presence of butyl xanthate and other reagents. This suggests that the gas species are involved in the wetting and flotation processes in conjunction with other phenomena, particularly, oxidation of metal sulfides and adsorption of hydrophobizing agents, which control their hydrophilic/hydrophobic properties. We may hypothesize that the nanobubbles with larger height promote breakage of the interfacial water film upon collision of a mineral particle and an air bubble, while gas nanodomains covering a substantial surface area promote mineral surface hydrophobicity and subsequent spreading the solid/air interface, and so attachment of the particle to the large air bubble [19,44,45,46]. Some hints on this were found using AFM (Figure 1 and Figure 2, and Refs. [18,21,22]) and cryo-XPS [19,20]. The formation of nanobubbles on essentially oxidized hydrophilic surfaces seems to be suppressed. It is also possible that the TD-induced gas nanobubbles on the surfaces with a considerable amount of oxidation products or/and ultrafine particles produced by grinding [30] form a sort of surface hydrophilic “foam” [47] which decreases the flotation. These phenomena shedding light on the fundamentals of wetting and hydrophobicity in various applications need to be studied in more detail. 

It was found here that the flotation performance of the Pb-Zn sulfide ore can be significantly improved imposing conditions favorable for the generation of surface nanobubbles in the system immediately related to the mineral engineering practice. Phase separation and heating the ore and cooling the large volumes of water are energy-consuming, and would unlikely be utilized at operating plants directly. However, these effects should be taken into consideration when handling heated milling products or circulating water, in particular, at Gorevskoye mill situated in a cold winter climate zone. 

## Supplementary Materials

The following are available online at https://www.mdpi.com/2079-4991/10/7/1362/s1, Figures S1 and S2: additional AFM images of HOPG and PbS surfaces. Figure S3: photographs of the sessile water droplets cooled to 0.2 °C and placed on mineral plates, Figures S4 and S5: schemes of Gorevskoye Pb-Zn sulfide ore flotation in different regimes, Tables S1–S5: results of the bulk sulfide flotation of Gorevskoye Pb-Zn ore for different schemes.
