# Supplementary material for: Towards Understanding the Role of Surface Gas Nanostructures: Effect of Temperature Difference Pretreatment on Wetting and Flotation of Sulfide Minerals and Pb-Zn Ore
Source: Nanomaterials (Basel). 2020 Jul 12;10(7):1362. doi: 10.3390/nano10071362 (PMC7408013; doi:10.3390/nano10071362)
Supplement: Supplementary file 1 [file nanomaterials-10-01362-s001.pdf]

Supplementary Materials

Towards understanding the role of surface gas nanostructures: Effect of temperature difference pretreatment on wetting and flotation of sulfide minerals and Pb-Zn ore

Yuri Mikhlin *, Anton Karacharov, Sergey Vorobyev, Alexander Romanchenko, Svetlana Antsiferova, Svetlana Markosyan, and Maxim Likhatski

Institute of Chemistry and Chemical Technology, Krasnoyarsk Science Center of the Siberian Branch of the Russian Academy of Sciences, Akademgorodok, 50/24, Krasnoyarsk, 660036, Russia

* Correspondence: yumikh@icct.ru

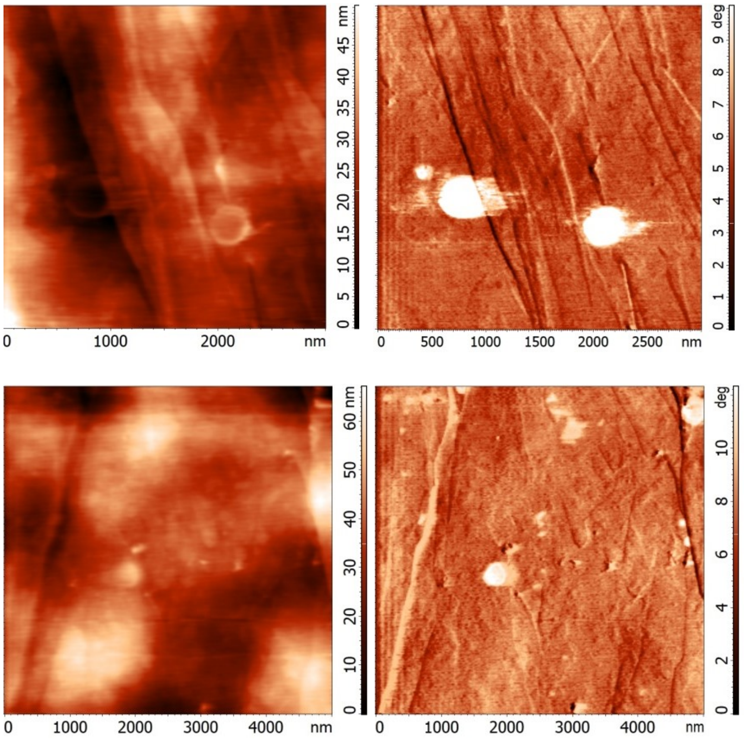

Figure S1. Examples of Tapping mode AFM height (left panes) and phase (right panels) images of HOPG surface heated to 50 °C and then conditioned in cold water (5 °C) before AFM experiment in ambient water.

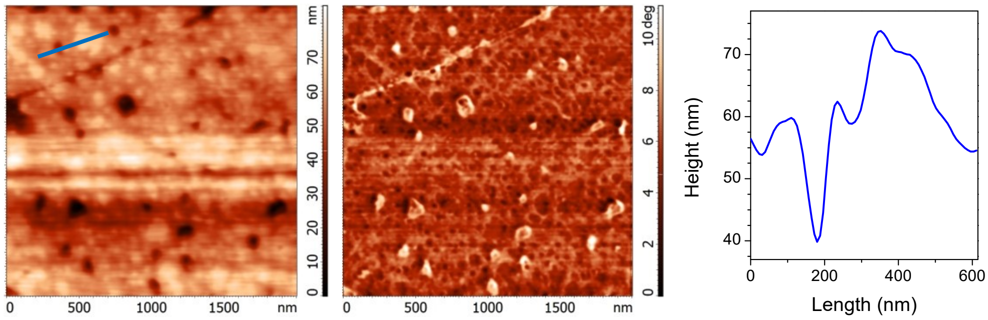

Figure S2. Tapping mode AFM images taken in water from galena PbS heated in air to 50 °C for 10 min and contacted with 10 mM KBX solution cooled down to 5 °C: height (left panel), phase (central) and the relief profile along the blue line at the height image.

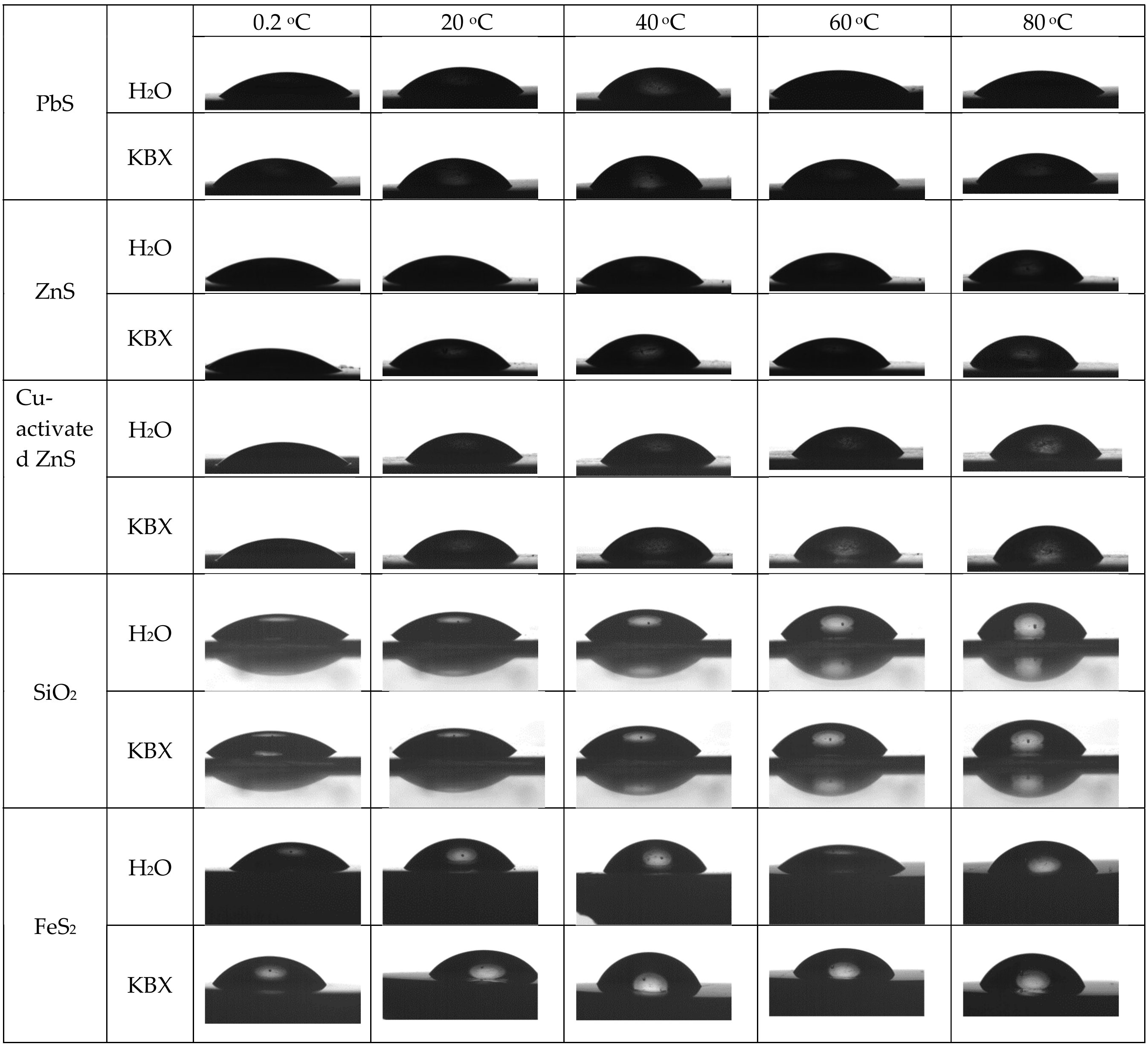

Figure S3. Photographs of the sessile water drops cooled to 0.2 °C and placed on the plates of galena, PbS, sphalerite, ZnS, pyrite, FeS_2, and SiO_2 rinsed with water (marked as H_2O), and preliminary reacted with 10 mM potassium n-butyl xanthate (KBX) solution for 10 min, kept at various temperatures. The drops on sphalerite activated with 0.1 mM $CuSO_4$ solution are also presented.

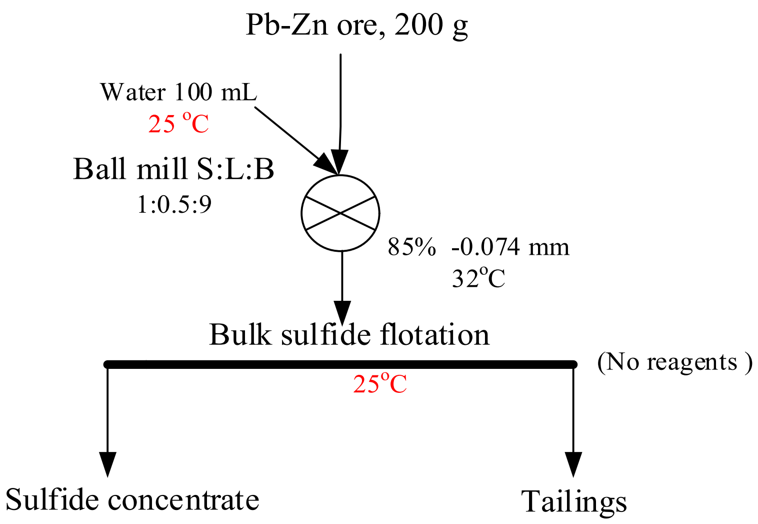

Figure S4. Scheme of flotation of Gorevskoye Pb-Zn sulfide ore in reagent-less regime; no temperature difference treatment.

Table S1. Results of flotation of Gorevskoye Pb-Zn sulfide ore in reagent-less regime without temperature difference treatment

Product	Yield, %	Content, wt.%		Recovery, %		Remarks
		Pb	Zn	Pb	Zn	
Bulk sulfide concentrate	1.38	22.95	3.04	7.54	1.63	No reagents, control test at ambient t°
Tailings	98.62	3.94	2.57	92.46	98.37	
Ore feed	100	4.2	2.58	100	100	

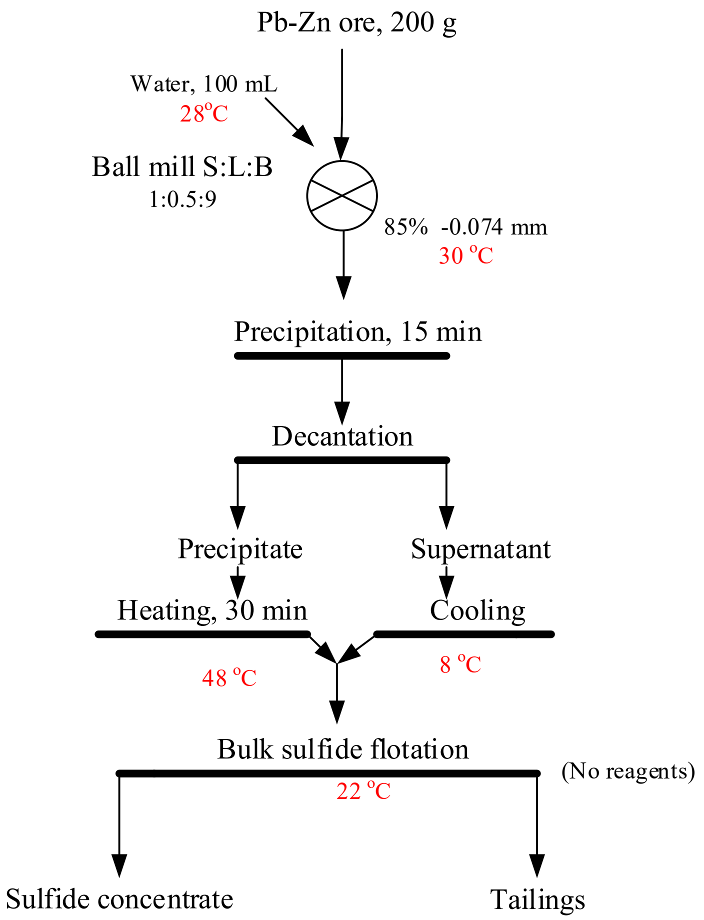

Figure S5. Flotation scheme of Gorevskoye Pb-Zn sulfide ore in reagentless regime with preliminary heating the ore and cooling water.

Table S2. Results of flotation of Gorevskoye Pb-Zn sulfide ore in reagentless regime with preliminary heating the ore and cooling water.

Product	Yield, %	Content, wt.%		Recovery, %		Remarks
		Pb	Zn	Pb	Zn	
Bulk sulfide concentrate	1.07	13.85	2.98	3.61	1.22	Residue heated, Water cooled
Tailings	98.93	4.0	2.62	96.39	98.78	
Ore feed	100	4.1	2.62	100	100	

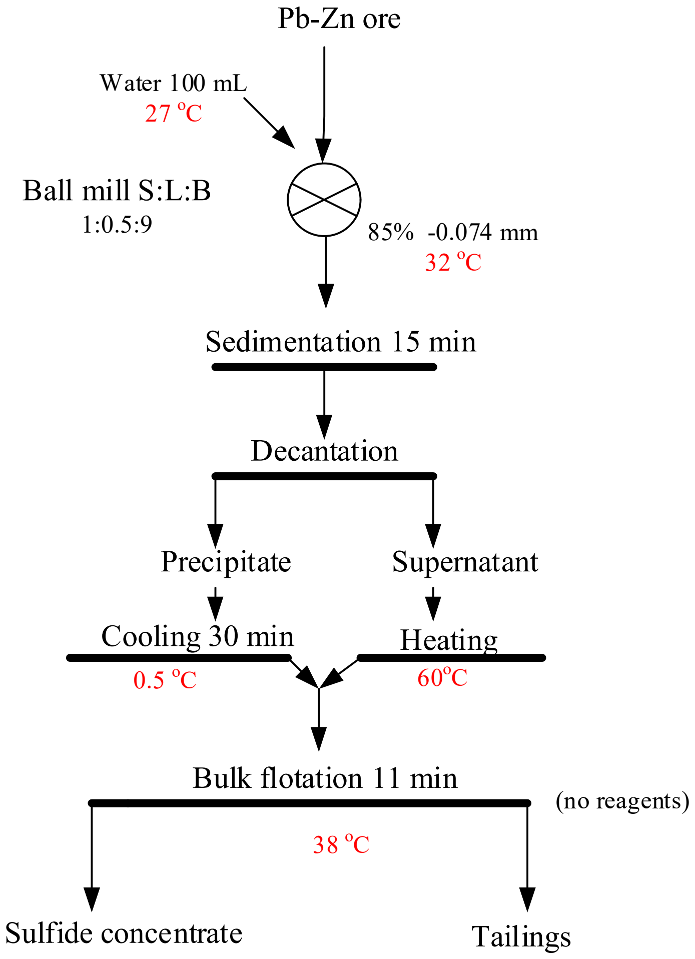

Figure S6. Scheme of flotation of Gorevskoye Pb-Zn sulfide ore in reagentless regime with preliminary cooling the ore and heating water.

Table S3. Results of the bulk sulfide flotation of Gorevskoye Pb-Zn sulfide ore in reagentless regime with preliminary heating the ore and cooling water.

Product	Yield, %	Content, wt.%		Recovery, %		Remarks
		Pb	Zn	Pb	Zn	
Bulk sulfide concentrate	1.68	35.4	3.15	14.3	2.04	Residue cooled,
Tailings	98.32	3.62	2.59	85.68	97.96	water heated
Ore feed	100	4.15	2.6	100	100	

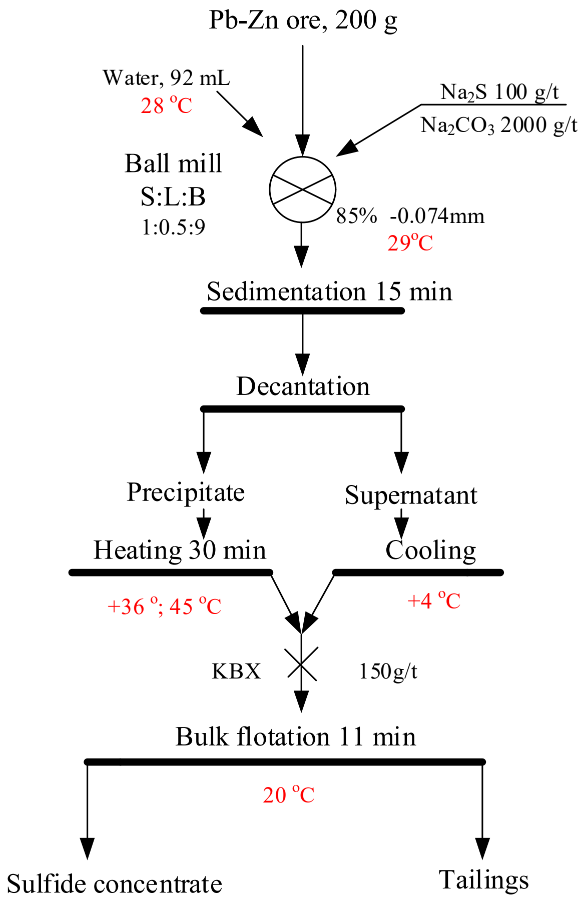

Figure S7. Typical scheme of flotation of Gorevskoye Pb-Zn sulfide ore with the dosage of butyl xanthate.

Table S4. Results of the bulk sulfide flotation of Gorevskoye Pb-Zn sulfide ore with butyl xanthate dosage.

Product	Yield, %	Content, wt.%		Recovery, %		Remarks
		Pb	Zn	Pb	Zn	
Bulk sulfide concentrate	7.11	30.8	5.65	58.39	14.45	KBX collector
Tailings	92.89	1.68	2.56	41.61	85.55	Control test at ambient t°
Ore feed	100	3.75	2.78	100	100	

Product	Yield, %	Content, wt.%		Recovery, %		Remarks
		Pb	Zn	Pb	Zn	
Sulfide concentrate	7.99	39.18	6.92	67.61	19.13	KBX collector
Tailings	92.01	1.63	2.54	32.39	80.87	Precipitate heated
Ore feed	100	4.63	2.89	100	100	to 36°
Sulfide concentrate	8.29	40.15	6.56	70.2	19.61	KBX collector
Tailings	91.71	1.54	2.43	29.8	80.39	Precipitate heated
Ore feed	100	4.74	2.77	100	100	to 45 °

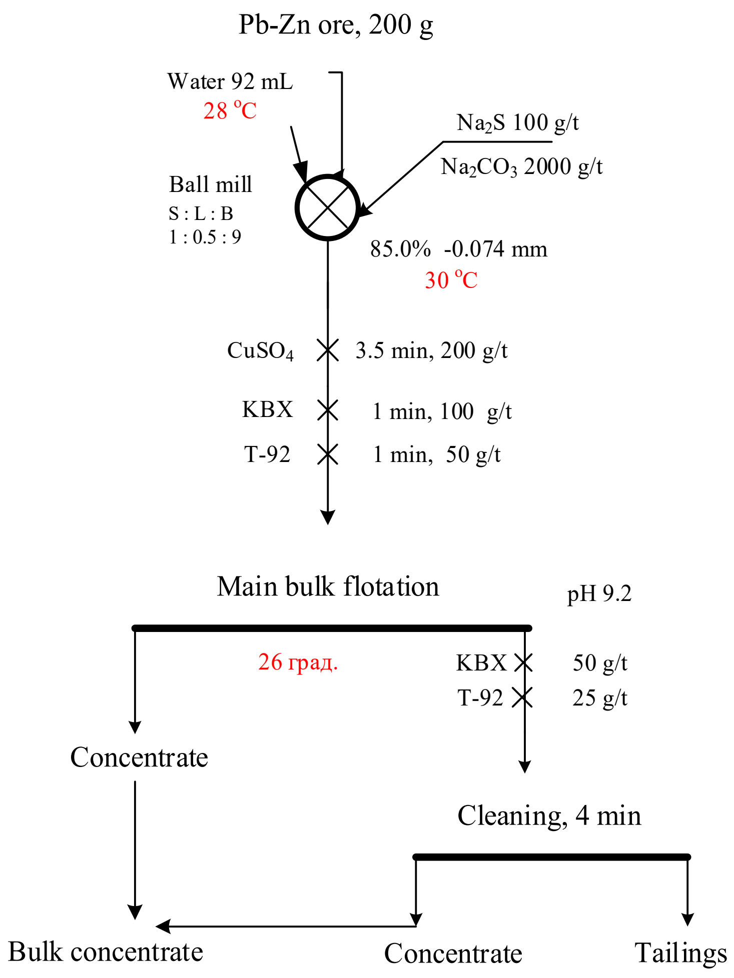

Figure S8. Scheme of flotation of Gorevskoye Pb-Zn sulfide ore with the dosage of butyl xanthate and copper sulfate; no temperature difference treatment.

Table S5. Results of the bulk sulfide flotation of Gorevskoye Pb-Zn ore with the dosage of butyl xanthate and copper sulfate; no temperature difference treatment applied.

Product	Yield, %	Content, wt.%		Recovery, %		Remarks
		Pb	Zn	Pb	Zn	
Sulfide concentrate	9.89	26.8	20.6	65.89	78.16	Collector, $CuSO_4$
Concentrate from cleaning	3.6	14.8	7.4	13.25	10.22	Control test at ambient t°
Bulk sulfide concentrate	13.49	23.6	17.08	79.14	88.37	
Tailings	86.51	0.97	0.35	20.86	11.62	
Ore feed	100	4.02	2.61	100	100	

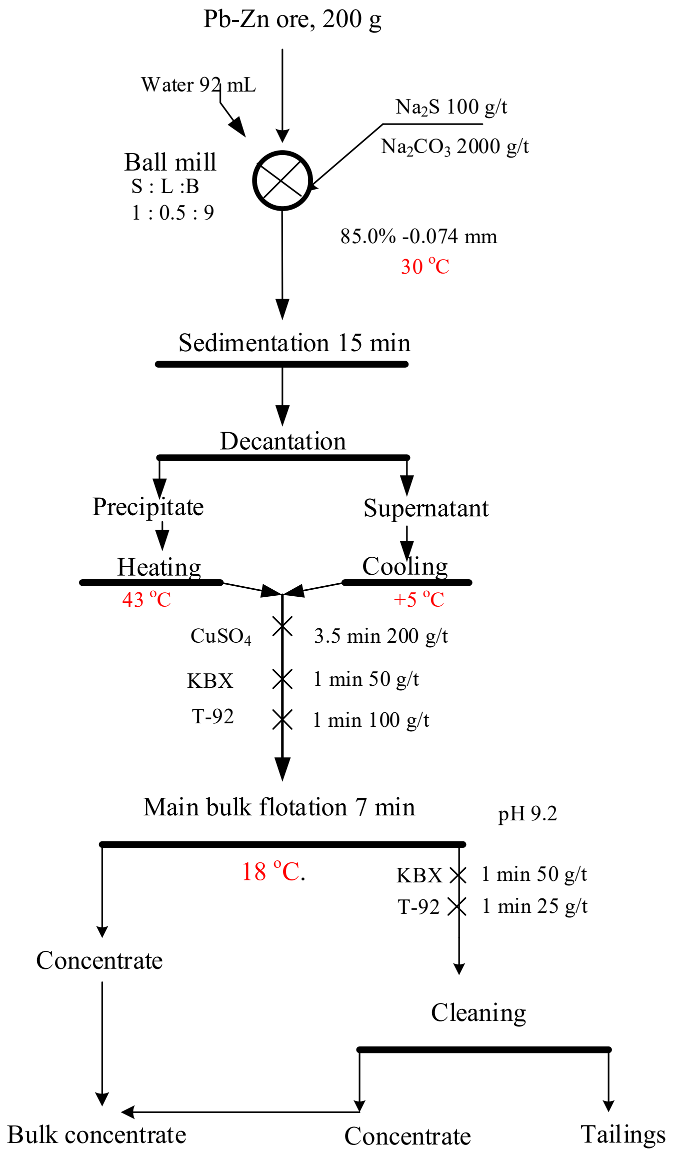

Figure S9. Scheme of flotation Gorevskoye Pb-Zn sulfide ore with the dosage of butyl xanthate and copper sulfate with the temperature difference pretreatment.

Table S6. Results of the bulk sulfide flotation of Gorevskoye Pb-Zn ore with the dosage of butyl xanthate and copper sulfate with the temperature difference pre-treatment applied.

Product	Yield, %	Content, wt. %		Recovery, %		Remarks
		Pb	Zn	Pb	Zn	
Sulfide concentrate	10.61	28.3	21.1	69.24	81.14	Collector, $CuSO_4$
Concentrate from cleaning	3.81	12.1	5.8	10.63	8.01	Residue heated to 43°
Bulk sulfide concentrate	14.42	24.02	17.06	79.87	89.15	water cooled to +5°
Tailings	85.58	1.02	0.35	20.13	10.85	
Ore feed	100	4.34	2.76	100	100	
